# *Arabidopsis thaliana* mTERF proteins: evolution and functional classification

**DOI:** 10.3389/fpls.2012.00233

**Published:** 2012-10-15

**Authors:** Tatjana Kleine

**Affiliations:** Department Biology I, Plant Molecular Biology (Botany), Ludwig Maximilians UniversityMunich, Germany

**Keywords:** mTERF function, organellar gene expression, chloroplasts, mitochondria, nucleus, retrograde signaling

## Abstract

Organellar gene expression (OGE) is crucial for plant development, photosynthesis, and respiration, but our understanding of the mechanisms that control it is still relatively poor. Thus, OGE requires various nucleus-encoded proteins that promote transcription, splicing, trimming, and editing of organellar RNAs, and regulate translation. In metazoans, proteins of the mitochondrial Transcription tERmination Factor (mTERF) family interact with the mitochondrial chromosome and regulate transcriptional initiation and termination. Sequencing of the *Arabidopsis thaliana* genome led to the identification of a diversified *MTERF* gene family but, in contrast to mammalian mTERFs, knowledge about the function of these proteins in photosynthetic organisms is scarce. In this hypothesis article, I show that tandem duplications and one block duplication contributed to the large number of *MTERF* genes in *A. thaliana*, and propose that the expansion of the family is related to the evolution of land plants. The *MTERF* genes—especially the duplicated genes—display a number of distinct mRNA accumulation patterns, suggesting functional diversification of mTERF proteins to increase adaptability to environmental changes. Indeed, hypothetical functions for the different mTERF proteins can be predicted using co-expression analysis and gene ontology (GO) annotations. On this basis, mTERF proteins can be sorted into five groups. Members of the “chloroplast” and “chloroplast-associated” clusters are principally involved in chloroplast gene expression, embryogenesis, and protein catabolism, while representatives of the “mitochondrial” cluster seem to participate in DNA and RNA metabolism in that organelle. Moreover, members of the “mitochondrion-associated” cluster and the “low expression” group may act in the nucleus and/or the cytosol. As proteins involved in OGE and presumably nuclear gene expression (NGE), mTERFs are ideal candidates for the coordination of the expression of organelle and nuclear genomes.

## Introduction

Chloroplasts and mitochondria are derived from progenitors that resembled cyanobacteria and extant α-proteobacteria, respectively, and have lost most of their ancestral genes to the nucleus of their eukaryotic host. The reduced genomes they have retained predominantly code for proteins involved in energy production and organellar gene expression (OGE) (Leister and Kleine, 2011). Hence tight coordination of nuclear gene expression (NGE) with OGE is required for the development of functional organelles. Although both types of organelle still display features of prokaryotic gene expression, their OGE systems are far more complex than those of their progenitors (Liere et al., 2011). Thus, mature organellar RNAs and proteins are generated by the concerted action of a plethora of nucleus-encoded proteins acquired or recruited during plant evolution, comprising additional RNA polymerases and sigma factors, and mono- or mero-specific RNA maturation factors that promote transcription, RNA splicing, editing, end formation, or translation (Maier et al., 2008; Stern et al., 2010; Barkan, 2011; Liere et al., 2011). This complexity explains why our understanding of the mechanisms controlling OGE is still relatively poor.

Sequencing of the *Arabidopsis thaliana* genome (Arabidopsis Genome Initiative, 2000) led to the identification of many novel gene families, among them the mitochondrial Transcription tERmination Factor (mTERF) family. In mammals, mTERFs were recognized a quarter of a century ago with the identification of mTERF1 as a factor that promoted transcription termination in human mitochondrial extracts (Kruse et al., 1989). The mTERF family in both metazoans and plants consists of four subfamilies named mTERF1–4 (Linder et al., 2005; Roberti et al., 2009). mTERF proteins have a modular architecture characterized by the repetition of a 30-amino acid motif named the MTERF motif. The number and disposition of these motifs, as well as the remaining sequences, vary widely within the family (http://smart.embl-heidelberg.de/).

In animals, mTERF proteins interact with the mitochondrial chromosome and regulate transcription by intervening in both termination and initiation (Park et al., 2007; Wenz et al., 2009). In mouse, mTERF3 and mTERF4 are required for embryo development (Park et al., 2007; Camara et al., 2011). While mTERF4 controls mitochondrial ribosomal biogenesis and translation by recruiting an rRNA methyltransferase to the large ribosomal subunit (Camara et al., 2011), mTERF3 binds to the promoter region of mitochondrial DNA and acts as a negative regulator of transcriptional initiation on both strands (Park et al., 2007). Furthermore, in metazoans, mTERF proteins act as genuine transcription termination factors (Kruse et al., 1989; Asin-Cayuela et al., 2005), and the recently published structure of human mTERF1 bound to DNA provides detailed insight into the mechanism of transcription termination in the mitochondrion (Jimenez-Menendez et al., 2010; Yakubovskaya et al., 2010). Moreover, *in vitro* interaction studies suggest that mitochondrial DNA mediates interactions between different mTERF proteins (Wenz et al., 2009). Thus, mTERF proteins fulfill diverse roles in mitochondrial gene expression, and multifunctionality and interdependency seem to be hallmarks of the family in animals.

Plants contain far more mTERFs than mammals (*A. thaliana* and *Oryza sativa* Japonica contain at least 35 and 48 mTERF proteins, respectively; http://smart.embl-heidelberg.de/), but knowledge about their function in photosynthetic organisms is sparse, and so far only four plant mTERF proteins have been functionally characterized (Schönfeld et al., 2004; Meskauskiene et al., 2009; Babiychuk et al., 2011; Mokry et al., 2011; Quesada et al., 2011). Because most *A. thaliana* mTERF proteins are targeted to mitochondria and chloroplasts (Babiychuk et al., 2011), elucidating the function of mTERF proteins promises to reveal important facets of the interaction between the nucleus and organelles in plants.

In this hypothesis article, I briefly summarize the information currently available on mTERF proteins in photosynthetic organisms. Bioinformatic analyses imply that the mTERF family has expanded during the evolution of land plants, and mTERF proteins have undergone functional diversification. Indeed, the function of mTERF proteins in plants appears not to be limited to organelles, and the most likely candidates for nuclear and/or cytosolic roles are identified and discussed.

## Emerging roles for mTERF proteins in plants

In *Chlamydomonas*, loss of *MOC1* (mTERF-like protein of *Chlamydomonas* 1), which is targeted to mitochondria, causes sensitivity to high light and disrupts transcription of genes for subunits of mitochondrial respiratory complexes (Schönfeld et al., 2004). Similarly, the *A. thaliana* mutant *soldat10*, the first *mterf* mutant to be characterized in higher plants, suffers from mild photo-oxidative stress, while complete inactivation of *SOLDAT10* (*AT2G03050*) is apparently lethal (Meskauskiene et al., 2009). SOLDAT10 is localized to chloroplasts, and the *soldat10* mutation seems selectively to affect levels of 16S and 23S rRNAs. The consequent drop in rates of protein synthesis in plastids activates retrograde signaling to the nucleus (Meskauskiene et al., 2009). The *A. thaliana* mTERF protein AT4G02990, variously named BELAYA SMERT (BSM; Babiychuk et al., 2011) or RUGOSA2 (RUG2; Quesada et al., 2011), has a broader function, and is essential for normal plant development, being required for the maintenance of the correct levels of transcripts in both mitochondria and chloroplasts (Babiychuk et al., 2011; Quesada et al., 2011). In the *bsm* mutant the processing of plastid transcripts is affected and embryo development is prematurely arrested (Babiychuk et al., 2011). The *twirt1* (*twr-1; at5g55580*) mutant shows reduced root growth and delayed shoot meristem activation, which results from a large reduction in the volume of shoot apical meristem in mature mutant embryos (Mokry et al., 2011). Furthermore, mTERF proteins have been shown to be associated with the plastid transcriptionally active chromosome (pTAC) in *A. thaliana* (Pfalz et al., 2006) and are found in nucleoid-enriched proteomes of maize (Majeran et al., 2011). As the nucleoid-enriched proteome includes proteins involved in DNA replication, organization and repair, as well as transcription, mRNA processing, splicing, and editing, this argues for a pivotal role of mTERF proteins in OGE (Majeran et al., 2011).

## Distribution of genes encoding mTERF proteins in plants

**Figure 1:**
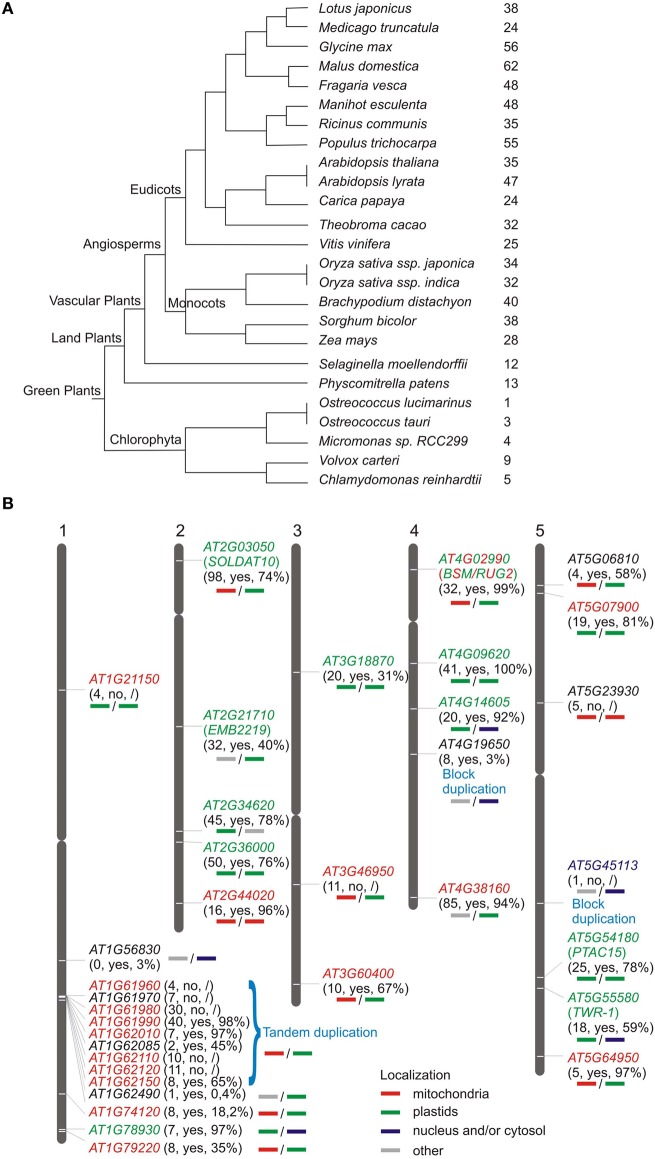
**Distribution of genes encoding mTERF proteins in plants. (A)** Numbers of *MTERF* genes in sequenced green plants according to the PLAZA database (http://bioinformatics.psb.ugent.be/plaza/). **(B)** Chromosomal distribution of *A. thaliana MTERF* genes. Approximate positions of *MTERF* genes are displayed on the respective chromosome. The first numbers in parentheses report the EST support values; “yes” or “no” indicates whether or not the respective *MTERF* is represented on Affymetrix ATH1 arrays; the percentage value indicates the fraction of all microarray datasets deposited in the Genevestigator database (https://www.genevestigator.com/gv/; Hruz et al., 2008) that record the presence of transcripts of the various *MTERF* genes. Predicted localizations for the different mTERF proteins were inferred from WolF PSORT (http://wolfpsort.org/) and TargetP (http://www.cbs.dtu.dk/services/TargetP/) scores (bold lines under the respective *MTERF* genes); the experimentally determined localizations for all mTERF proteins (Babiychuk et al., 2011) and for AT4G02990 (Quesada et al., 2011) are indicated in colored lettering.

Although mTERF proteins are widespread in metazoans (Roberti et al., 2009), their numbers are strikingly high in plants relative to other eukaryotes. For example, *Drosophila melanogaster*, *Mus musculus* and *Homo sapiens* each contain only four mTERF proteins (Linder et al., 2005), whereas *A. thaliana* and *O. sativa* Japonica contain at least 35 (Babiychuk et al., 2011) and 48 genes for mTERF proteins, respectively (Figure 1A; http://smart.embl-heidelberg.de/). Other plant species, such as *Zea mays*, *Vitis vinifera*, and *Populus trichocarpa* contain similarly high numbers of *MTERF* genes (Figure 1A). Since there are fewer putative mTERF proteins in lower plants—13 in *Physcomitrella patens*, a land moss, and six in the green alga *Chlamydomonas reinhardtii* (http://www.ebi.ac.uk/interpro/IEntry?ac=IPR003690)—the mTERF family presumably expanded during the evolution of land plants.

The genes encoding mTERF proteins from *A. thaliana* are distributed over all five chromosomes (Figure 1B). The lower arm of chromosome 1 is the most highly populated, with 14 *MTERF* genes, whereof a 70 kb region contains nine *MTERF* genes formed from tandem duplications (local duplications that often result from unequal crossing over). According to the PLAZA database (http://bioinformatics.psb.ugent.be/plaza/; Van Bel et al., 2012), *AT4G19650* and *AT5G45113* are segmental (or also called block) duplicates which result from large scale genome events such as polyploidy or duplications of large chromosomal regions.

## mRNA expression patterns of *MTERF* genes in *A. thaliana*

Of the 1,435,214 EST (expressed sequence tag) sequences deposited in the AtGDB (*A. thaliana* Genome Data Base; http://www.plantgdb.org/XGDB/phplib/resource.php?GDB=At), 668 are assigned to *MTERF* genes, which corresponds to an average of 20 ESTs per *MTERF* gene (in comparison to an overall average of 42.7 ESTs per *A. thaliana* gene). *AT1G56380* is not represented by an EST, *AT1G62490* and *AT5G45113* (a block duplicate) have only one EST each, and *AT1G21150*, *AT1G61960*, *AT1G62085*, *AT5G06810*, *AT5G54180*, and *AT5G64950* are represented by ≤ 5 each (Figure 1B). These figures indicate that *MTERF* genes are expressed at low to medium levels and suggest that *AT1G563890*, and perhaps *AT1G62490* and *AT5G45113*, are not expressed at all or alternatively are expressed in specific cell types or developmental stages that were not sampled yet. To investigate this further, the Genevestigator database (Hruz et al., 2008; https://www.genevestigator.com/gv/), which provides gene expression values for *A. thaliana* based on available Affymetrix GeneChip data, was consulted to determine the percentage of microarray experiments in which transcripts of the various *MTERFs* were detected (Figure 1B). Nine of the 35 *MTERF* genes could not be assessed as they are not represented on the Affymetrix ATH1 genome array (Figure 1B). The average fraction of present calls for transcripts of the remaining 26 *MTERF* genes is 65.5%, and transcripts with EST support of ≤ 1 are found in only 3% (in the case of *AT1G56380*) and 0.4% (in the case of AT1G62490) of the samples tested on microarrays (Figure 1B). Taken together, these data suggest that most *MTERF* genes are actively transcribed.

**Figure 2:**
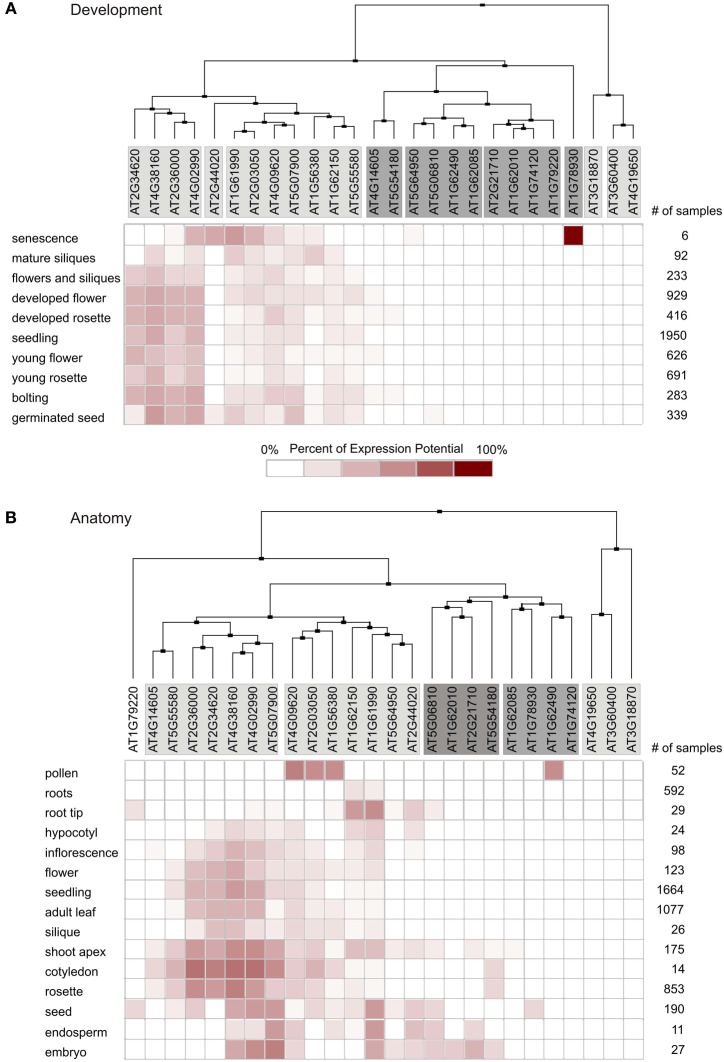
**Global expression profiling of *MTERF* genes in *A. thaliana*.** Expression profiles were determined with the Clustering Tool in Genevestigator. *MTERF* mRNA expression data are shown for different developmental stages **(A)** and different plant organs **(B)**. The cladogram at the top summarizes the degree of relatedness between the expression profiles of the different *MTERF*s. number of samples, number of microarrays covering the different categories.

The Genevestigator database was then used to perform global expression profiling of *MTERF* genes in *A. thaliana*. The clustering tools in Genevestigator group genes according to their expression potential (EP), which is defined as the average of the top 1% of signal values across all samples for a given probe set. Thus, the EP is a robust measure for the maximum expression level of any given gene. Hierarchical clustering with the Development Tool, which provides gene expression values for different developmental stages, revealed that four *MTERF*s (*AT2G34620*, *AT2G36000*, *AT4G02990*, and *AT4G38160*) are expressed at levels equivalent to 40–60% of EP in nearly all developmental stages (Figure 2A). A second cluster consisting of seven *MTERF*s reaches 20–40% of EP in nearly all phases of development. These values reflect their generally high EP and the high percentage of present calls on all arrays. Localized expression of *AT2G44020* and *AT1G78930* during senescence (Figure 2A) might point to a specific role for these two mTERFS in this process. Moreover, *AT2G44020* is expressed at 20% of EP in the germinated seed stage. The remaining *MTERF*s display a very low level of expression relative to EP over the whole life cycle.

Clustering with the Anatomy Tool shows that *MTERFs* are generally expressed at low levels in roots (Figure 2B). A group of 12 *MTERF* genes distributed in two clusters is expressed in nearly all other organs investigated (hypocotyl, cotyledons, leaves, flowers, seeds, embryo, and pollen). Only *AT1G62490* shows a notably higher relative level of expression in pollen (Figure 2B).

**Figure 3:**
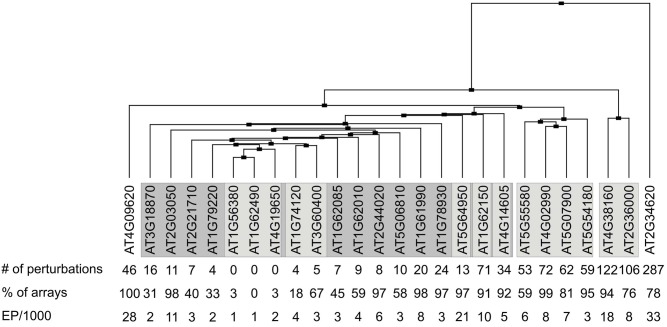
**Clustering of transcriptional responses of *A. thaliana MTERF* genes to different perturbations.** The Genevestigator Perturbations Tool was applied to all available *A. thaliana* microarrays in combination with the two-fold change filter and a *p*-value of < 0.05. The cladogram at the top summarizes the relatedness of *MTERF* responses. number of perturbations, number of perturbations (for example chemicals, hormones, stress and mutations) that evoke ≥ 2-fold changes in the levels of *MTERF* mRNAs; % of arrays, the value indicates the percentage of microarray datasets in Genevestigator that detected the presence of transcripts of the various *MTERF* genes; the expression potential (EP) was divided by 1000.

To assess to what extent *MTERF* transcripts are regulated in response to various experimental perturbations (exposure to chemicals, hormones or stress, and mutation of specific genes), the Genevestigator Perturbations Tool was applied to all available *A. thaliana* microarrays in conjunction with the two-fold change filter and a *p-*value of < 0.05. *AT2G34620* is most susceptible to perturbations: 287 conditions provoke at least a two-fold change in transcript level (Figure 3). Of all *MTERF*s, *AT2G34620* seems to be most strongly induced by light and is repressed by cold, drought, lincomycin (an inhibitor of organellar protein synthesis) and infection with *Phytophthora infestans* or *Pseudomonas syringae*. Other *MTERF* genes induced by light (particularly blue light) are *AT2G36000*, *AT4G02990*, *AT4G3810*, *AT5G07900*, and *AT5G55580*. The latter genes show similar expression profiles in response to perturbations (Figure 3) and, interestingly, they are also expressed together with *AT2G34620* in various organs (Figure 2B). In addition, *AT1G62150* and *AT4G09620* are moderately regulated in response to 71 and 46 conditions, respectively. In contrast, *AT1G74120* is induced only during germination and does not respond to other conditions, while *AT3G60400* is repressed only during germination and upon exposure to heat. Note that the responses revealed by the Perturbations Tool are based on comparisons between experimental and control samples from the same experiment. If the same condition is tested in several experiments, this condition will be scored multiple times. Thus, the number of perturbations impinging on expression of at least some *MTERF*s will be overestimated by the tool.

In sum, the members of the *A. thaliana MTERF* gene family show diverse mRNA expression patterns. Strikingly, duplicated genes do not display the same transcript accumulation patterns, suggesting that they have undergone functional diversification during evolution to increase adaptability to environmental changes.

## Predicting hypothetical functions of the different mTERF proteins using co-expression analysis

**Figure 4:**
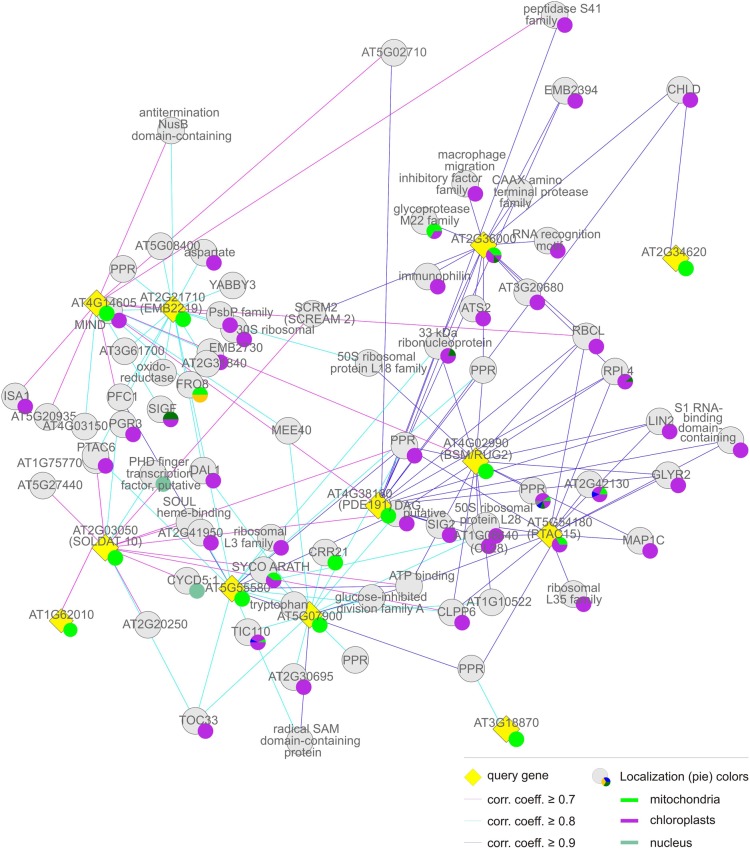
**Group (i), the “chloroplast” cluster.** Coregulation gene network derived from condition-independent co-expression analysis using the CORrelation NETworks tool (CORNET2.0; https://bioinformatics.psb.ugent.be/cornet; De Bodt et al., 2012). Only strong coregulations of the top 15 genes with a Pearson correlation coefficient (corr. coeff.) ≥ 0.7 are shown. The size of a localization pie represents how much a value is referenced compared to the others.

**Figure 5:**
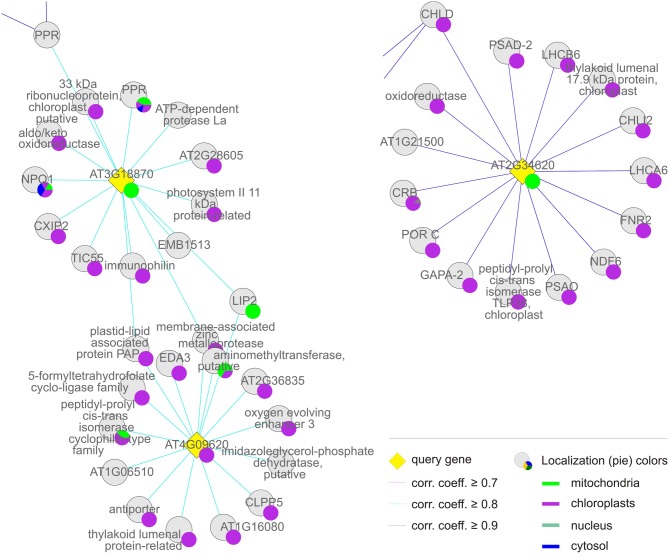
**Group (ii), the “chloroplast-associated” cluster.** The coregulation network was generated as in Figure 4.

**Figure 6:**
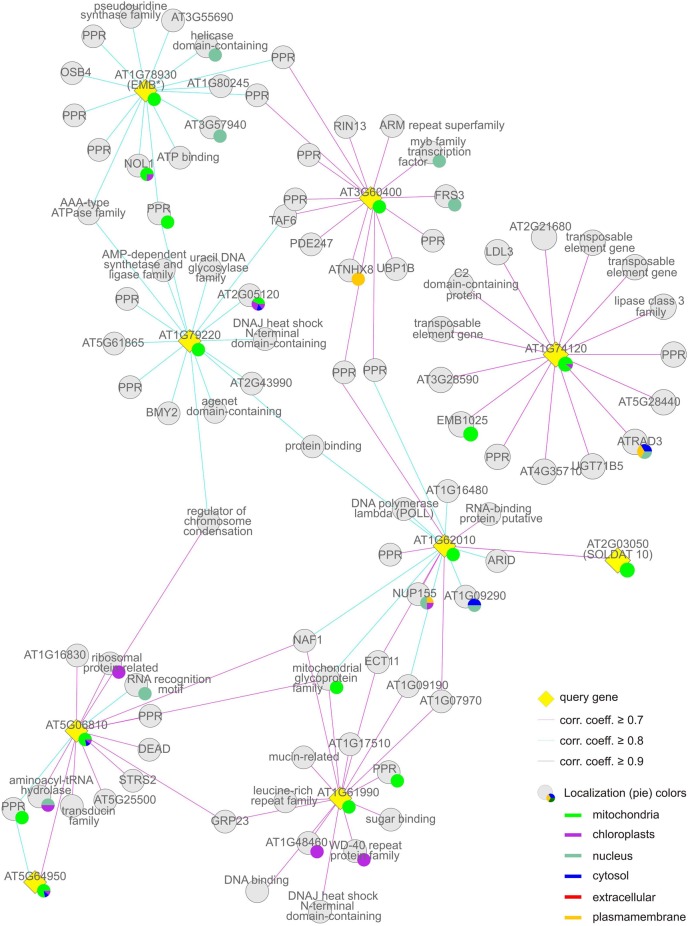
**Group (iii), the “mitochondrial” cluster.** The coregulation network was generated as in Figure 4.

**Figure 7:**
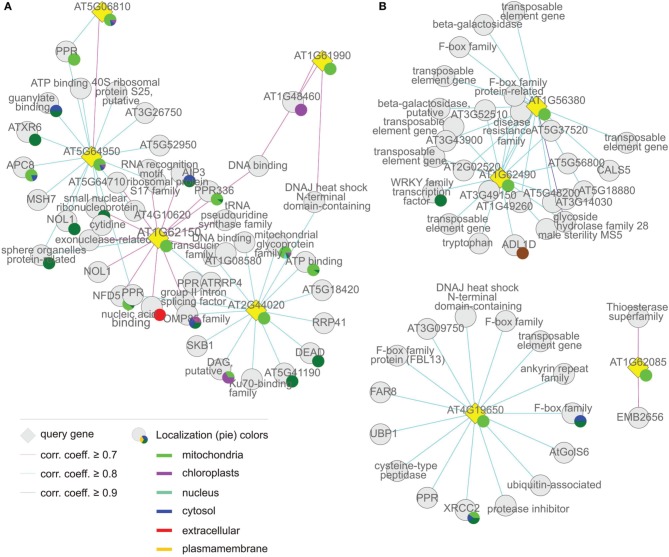
**Coregulation network of (A) group (iv), the “mitochondrion associated” cluster and (B) group (v), “the others.”** The coregulation network was generated as in Figure 4.

One way of generating hypotheses about genes whose functions are unknown is to identify genes of known function that are co-expressed with them across a wide range of tissues and treatments (Horan et al., 2008). This principle, the idea of “guilt-by-association” (Usadel et al., 2009), has already been successfully applied to pinpoint novel photosynthetic genes (DalCorso et al., 2008) in groups of transcriptionally coregulated nuclear genes (regulons) in *A. thaliana* in which photosynthetic genes were overrepresented (Biehl et al., 2005). Here, the CORrelation NETworks tool (CORNET2.0; https://bioinformatics.psb.ugent.be/cornet; De Bodt et al., 2012) was used to construct a condition-independent (based on the selection of all deposited microarray data; Usadel et al., 2009) co-expression network for the 26 *MTERF*s represented by probes on the Affymetrix ATH1 genome array (Figures 4–7). The CORNET2.0 tool uses improved genome annotation and slightly different parameter settings to follow the expression of 21,428 genes instead of 20,777 genes monitored before. Importantly, the *p*-values for a correlation coefficient are calculated on the basis of the correlation coefficient and the number of conditions in the expression dataset. Thus, the *p*-value is a better co-expression measure than the correlation coefficient when comparing results from different expression datasets. Therefore, co-expression analysis was performed with the top 15 genes whose Pearson correlation coefficients with a given *MTERF* were ≥ 0.7 and of a *p*-value ≤ 0.05, and the data were displayed in Cytoscape (Smoot et al., 2011). The resulting clusters, together with data pertaining to the subcellular localizations of the proteins encoded by the genes co-expressed with each *MTERF* defined by the Gene Ontology (GO) Annotations tool provided by The Arabidopsis Information Resource (TAIR) (http://arabidopsis.org/tools/bulk/go/index.jsp), were then used to sort the mTERFs into five groups, the members of which are listed in Table 1.

**Table 1:** **Overview of the different groups of mTERF proteins**.

**Name**	**AGI identifier**	**ChloroP/WolfPSort, experimental localization**	**Co-expression**
**GROUP (I), THE “CHLOROPLAST” CLUSTER**			
mTERF1/SOLDAT10^a^	AT2G03050	M/C, C	**C**, N, other
mTERF2/EMB2219^b^	AT2G21710^*^	O/C, C	**C**, M, other
mTERF3	AT2G36000^*^	C/C, C	all C
mTERF4/BSM^c^/RUG^d^	AT4G02990^*^	M/C, C	all C
mTERF5	AT4G14605^*^	C/Y, C	**C**, other
mTERF6	AT4G38160	O/C, M	all C
mTERF7	AT5G07900^*^	C/C, M	**C**, M
mTERF8/PTAC15^e^	AT5G54180	C/C, C	all C
mTERF9/TWIRT1^f^	AT5G55580^*^	C/Y, C	**C**, M
**GROUP (II), THE “CHLOROPLAST-ASSOCIATED” CLUSTER**			
mTERF10	AT2G34620	C/N, C	all C
mTERF11	AT3G18870	C/C, C	all C
mTERF12	AT4G09620	C/C, C	**C**, M
**GROUP (III), THE “MITOCHONDRIAL” CLUSTER**			
mTERF13	AT1G61990	M/C, M	M, C, **O**
mTERF14	AT1G62010	M/C, M	M, C, **O**
mTERF15	AT1G74120	M/C, M	M, C, **O**
mTERF16	AT1G78930^*^	C/N, C	M, C, **O**
mTERF17	AT1G79220	M/C, M	N, C, M, O
mTERF18	AT3G60400	M/C, M	M, C, N, O
mTERF19	AT5G06810	M/C, n.d.	M, C, N, O
**GROUP (IV), THE “MITOCHONDRION-ASSOCIATED” CLUSTER**			
mTERF20	AT1G62150	M/C, M	N, M, O
mTERF21	AT2G44020	M/M, M	M, C, N, Y, O
mTERF22	AT5G64950	M/C, M	N, Y, M, O
**GROUP (V), “THE OTHERS”**			
mTERF23	AT1G56380	O/N, n.d.	N, cell wall, Golgi, O
mTERF24	AT1G62085	M/C, n.d.	plasmodesmata, O
mTERF25	AT1G62490	O/C, n.d.	N, cell wall, Golgi, O
mTERF26	AT4G19650	O/Y, n.d.	O
**NOT REPRESENTED ON ATH1 AFFYMETRIX ARRAYS**			
mTERF27	AT1G21150^*^	C/C, M	
mTERF28	AT1G61960	M/C, M	
mTERF29	AT1G61970	M/C, n.d.	
mTERF30	AT1G61980	MC/M, M	
mTERF31	AT1G62110	M/C, M	
mTERF32	AT1G62120	M/C, M	
mTERF33	AT3G46950	M/C, M	
mTERF34	AT5G23930	M/M, n.d.	
mTERF35	AT5G45113	O/Y, N/Y	

a Meskauskiene et al., 2009;

b Tzafrir et al., 2004;

c Babiychuk et al., 2011;

d Quesada et al., 2011;

e Pfalz et al., 2006;

f Mokry et al., 2011;

* Homolog identified in maize nucleoids. Experimentally proven localizations (Babiychuk et al., 2011; Quesada et al., 2011) were compared to bioinformatics predictions from TargetP and WolF PSORT. Bold lettering indicates the organelle to which most of the co-expressed gene products are targeted. C, chloroplast; M, mitochondria; N, nucleus; O, others; Y, cytosol.

The *MTERF* genes that make up the first and largest set, the “chloroplast” group (*AT2G36000*, *AT4G02990*, *AT4G14605*, *AT4G38160*, *AT5G07900*, *AT5G54180*, *AT5G55580*, *AT2G03050*, and *AT2G21710*) themselves form a co-expression cluster (Figure 4, Tables 1 and 2), and their products (mTERF1–9) are known or predicted to reside in chloroplasts. The products of most non-*MTERF* genes belonging to this cluster are also localized in the chloroplast.

**Table 2:** **GO analysis of the genes co-expressed with the different groups of *MTERFs***.

**GO cellular component**	**Whole genome**	**Group (i)**	**Group (ii)**	**Group (iii)**	**Group (iv)**	**Group (v)**
Chloroplast	8.9	30.1 (338)	25.6 (287)	9.3 (105)	3.0 (33)	2.4 (27)
Plastid	4.3	15.9 (369)	17.8 (412)	3.7 (86)	4.5 (104)	0.0 (0)
Mitochondrion	2.8	1.4 (52)	0.5 (19)	15.0 (541)	11.9 (432)	2.4 (88)
Nucleus	5.6	1.4 (26)	0.0 (0)	6.5 (117)	10.4 (186)	0.0 (0)
Cytosol	3.7	0.6 (16)	0.5 (14)	0.9 (25)	11.9 (325)	0.0 (0)
Ribosome	1.9	3.2 (167)	0.5 (28)	1.9 (98)	3.0 (156)	0.0 (0)
Unknown cellular components	18.7	0.9 (5)	0.0 (0)	31.8 (170)	16.4 (88)	68.3 (365)
Other cellular components	8.5	0.3 (3)	0.5 (6)	3.7 (44)	3.0 (85)	17.1 (200)

The entries indicate the frequency of assignment (expressed as a percentage) of the listed GO (gene ontology) terms to the genes co-expressed with MTERF members of the respective groups (i–v). Numbers in parentheses reflect the percentage of enrichment compared to the whole genome list. GO annotations were determined with TAIR.

Group (ii), the “chloroplast-associated” set, comprises three *MTERF* genes (*AT2G34620*, *AT3G18870*, and *AT4G09620*) whose products (mTERF10–12) have been shown to be localized to chloroplasts. Nearly all co-expressed gene products also reside in the chloroplast. Group (ii) is considered as a discrete group, since members of this group do not cluster with those in group (i) and co-expressed genes encode more diverse functions (Figure 5, Tables 1 and 2).

The “mitochondrial” cluster comprises seven genes (*AT1G61990*, *AT1G62010*, *AT1G74120*, *AT1G78930*, *AT1G79220*, *AT3G60400*, and *AT5G06810*) that encode proteins (mTERF13–19) known or predicted to be located in mitochondria. Predicted localizations for the gene products co-expressed with them include mitochondria, chloroplasts, the nucleus and other compartments: These *MTERF* genes, with the exception of *AT1G74120*, themselves form a co-expression cluster (Figure 6, Tables 1 and 2).

The fourth “mitochondrion-associated” cluster has three members (*AT1G62150*, *AT2G44020*, *AT5G64950*), whose products (mTERF20–22) are known or predicted to be located in mitochondria or in the nucleus. Co-expressed proteins are found in mitochondria, the nucleus or other compartments. The *MTERF* genes in this group are defined as a discrete group since they form a single co-expression cluster different from the “mitochondrial cluster” (Figure 7A, Tables 1 and 2).

The fifth group, referred to here as “Others,” is made up of *AT1G56380*, *AT1G62085*, *AT1G62490*, and *AT4G19650*, the products of which (mTERF23–26) are predicted to be located in various compartments (Table 1), but attempts to localize corresponding GFP fusions have been unsuccessful (Babiychuk et al., 2011). Similarly, for the vast majority of co-expressed gene products, no definitive information on subcellular localization is yet available (Figure 7B, Tables 1 and 2).

### The chloroplast cluster: mTERF proteins involved in oge and embryogenesis

The nine *MTERF* genes in this group (Table 1) are broadly expressed; indeed, transcripts of most of them are reported in at least 75% of all array datasets in Genevestigator (Figure 1B). The values for *MTERF2* (*AT2G21710*) and *MTERF9* (*AT5G55580*) are 40 and 59%, respectively. Most members are expressed at all developmental stages and in all plant organs except roots (Figure 2B). Remarkably, all of the *A. thaliana* mTERF proteins so far described in the literature (Pfalz et al., 2006; Meskauskiene et al., 2009; Babiychuk et al., 2011; Mokry et al., 2011; Quesada et al., 2011)—SOLDAT10, BSM/RUG2, TWIRT1, and PTAC15—belong to this group. The embryo lethality of *soldat10* (Meskauskiene et al., 2009), *bsm* (Babiychuk et al., 2011), and *at2g21710* (*emb2219*) (Tzafrir et al., 2004) mutants, and the gametophyte lethality seen in the *mterf5* (*at4g14605*) mutant (Babiychuk et al., 2011), point to a role for this group of mTERF proteins in important developmental processes. Furthermore, of the eight mTERF proteins identified in maize nucleoids that show homology to *A. thaliana* mTERFs (Majeran et al., 2011), six belong to this group (Table 1), again suggesting that these mTERF proteins are critical for chloroplast gene expression (Figure 2A). This assumption is also supported by functional enrichment analyses. Thus, the TAIR GO Annotations Tool shows that genes for proteins localized to chloroplasts and plastid ribosomes are enriched 3.5-fold and 1.7-fold, respectively, in this co-expression cluster (relative to the genome as a whole), while genes for mitochondrial, nuclear, and cytosolic proteins are 52, 26, and 15% underrepresented (Table 2).

A more refined analysis, which only displays GO terms that are significantly over- or under-represented in sets of genes compared to the whole genome (Al-Shahrour et al., 2004), was performed with FatiGO (http://www.fatigo.org). Inspection of the GO terms for biological processes shows that, with TRANSLOCON AT THE INNER ENVELOPE MEMBRANE OF CHLOROPLASTS 110 (Tic110, AT1G06950), ACCUMULATION AND REPLICATION OF CHLOROPLASTS 11 (ARC11, AT5G24020), and a Clp protease subunit (ClpP6, AT1G11750), the category “plastid organization” (GO:0009657) is enriched 29-fold. Interestingly, all known substrates of ClpP6 function in more general housekeeping roles, such as plastid protein synthesis, folding, and quality control, rather than in metabolic activities such as photosynthesis (Sjögren et al., 2006).

The FatiGO analysis also reveals a 4-fold enrichment (*p*-value ≤ 0.05) for the cellular component category “ribonucleoprotein complex” (GO:0030529). Co-expressed genes assigned to this category code for RSRP-3 (AT5G15760), L3 (AT2G43030), L18p/L5e (AT3G20230), L28 (AT2G33450), and L35 (AT2G43030), all of which are members of the chloroplast ribosomal protein family. Moreover, EMBRYO DEFECTIVE 2784 (EMB2784, RPL4; AT1G07320), one of the RRM/RBD/RNP family of RNA-binding proteins (AT1G01080) and the rRNA-binding protein EMB2394 (AT1G05190) fall into this category. Moreover, several other co-expressed proteins important for chloroplast gene expression are involved in rRNA processing. These are DIFFERENTIATION AND GREENING-LIKE 1 (DAL1, *AT2G33430*), which is required for the maturation of plastid ribosomal RNAs and essential for chloroplast differentiation (Bisanz et al., 2003), and RIBONUCLEOTIDE REDUCTASE 1 (RNR1, EMB2730, AT5G02250), which codes for a 3–5′ exoribonuclease (Bollenbach et al., 2005). Another gene co-expressed with these *MTERF* genes is (*PTAC6*, *AT1G21600*). PTAC6 might also be involved in post-transcriptional processes, such as RNA processing and/or mRNA stabilization, and the *ptac6* mutant is inviable in the absence of exogenous carbon sources (Pfalz et al., 2006). In addition, with sigma subunits 2 and 6 of the plastid RNA polymerase (*SIG2* and *SIG6*, *AT1G08540*, and *AT2G36990*), central regulators of transcription are co-expressed with these *MTERF*s. It is also interesting to note that genes encoding two members of the tetratricopeptide repeat (TPR) family (TPR; *AT1G02150*, *AT2G18940*) and five pentatricopeptide repeat (PPR) proteins (AT2G17033, AT5G46580, AT5G55740, AT3G53700, and AT4G31850) families are co-expressed with MTERF1–9. The PPR family has approximately 450 members, and the PPR and TPR motifs are closely related (Small and Peeters, 2000). The majority of PPR and TPR proteins in *A. thaliana* are targeted to mitochondria and/or chloroplasts, where they have been proposed to function in the processing, editing, stabilization, and translation of RNA molecules (Lurin et al., 2004; Saha et al., 2007; Schmitz-Linneweber and Small, 2008).

Thus, taken together, these results strongly argue that the mTERF proteins in this group are mainly involved in (post)-transcriptional processes in organelles, primarily in the chloroplast, at all stages of development.

### The chloroplast-associated group: mTERF proteins involved in gene expression and/or protein catabolism in chloroplasts

According to TAIR, the group of gene products co-expressed with *MTERF10–12* is enriched (3.2-fold) for proteins located to chloroplasts or plastids, while proteins with mitochondrial, nuclear, cytosolic, and ribosomal localization (at 19, 0, 14, and 28%, respectively) are even more underrepresented than in group (i). Moreover, FatiGO analysis revealed a 27-fold enrichment (*p*-value ≤ 0.05) for the cellular component category “thylakoid” (GO:0009579). Included in this category are NON-PHOTOCHEMICAL QUENCHING 1 (NPQ1, AT1G08550), a violaxanthin deepoxidase involved in the xanthophyll cycle, and chloroplast proteins involved in protein folding (AT3G15520, AT1G18170, AT1G74070), a finding that is reflected in the significant enrichment for the molecular function category “peptidyl-prolyl *cis-trans* isomerase activity” (GO:0003755). The *MTERF* gene *AT2G34620* is linked to group (i) insofar as it is also co-expressed with *MG-CHELATASE SUBUNIT D* (*CHLD*, *AT1G08520*; Figure 5). Indeed, the biological process category “tetrapyrrole biosynthesis” (GO:0033014) is also overrepresented in group (ii), together with the category “photosynthesis” (GO:0015979); genes assigned to both categories are 36-fold enriched relative to the whole genome. In addition, a more diverse group of chloroplast genes is co-expressed with *MTERF10–12*, encoding a Psb27 homolog involved in PSII biogenesis (low PSII accumulation 19, LPA19, and AT1G05385), the plastid-lipid-associated protein PAP (AT2G35490), GLYCERALDEHYDE 3-PHOSPHATE DEHYDROGENASE A SUBUNIT 2 (GAPA-2, AT1G12900), a member of the NAD(P)-linked oxidoreductase superfamily (AT2G27680) and TRANSLOCON AT THE INNER ENVELOPE MEMBRANE OF CHLOROPLASTS 55 (Tic55, AT2G24820). Although the co-expressed genes make up a functionally diverse group of proteins, several of them are involved in protein binding and proteolysis. EMBRYO SAC DEVELOPMENT ARREST 3 (EDA3, AT2G34860) functions in the unfolded protein and heat shock responses and is involved megagametogenesis, while Clp PROTEASE 5 (ClpP5, AT1G02560), an ATP-dependent protease La (LON) domain protein (AT1G19740), and a peptidase M50 family protein (AT1G05140) all mediate protein degradation. Furthermore, with the RRM/RBD/RNP family member AT2G35410, chloroplast RNA-binding protein (CRB, CSP41B, AT1G09340), a TPR (AT1G01970) and two PPR proteins (AT1G12250 and AT2G17033), RNA-binding proteins are again well represented.

Thus, *MTERF* genes in this group are mostly co-expressed with photosynthesis genes and genes encoding chloroplast proteins of diverse function (this holds especially for the *MTERF* gene *AT2G34620*). However, genes which encode proteins that function in chloroplast gene expression and protein catabolism are also expressed in association with this group.

### The mitochondrial cluster: mTERF proteins involved in mitochondrial DNA and RNA metabolism

The set of genes co-expressed with the second largest group of *MTERF* genes (*MTERF13–19*; Table 1) is enriched 5.4-fold for sequences that encode mitochondrial proteins, while structural genes for proteins with a chloroplast or nuclear localization show no enrichment, and proteins predicted to reside in the cytosol are underrepresented (Table 2). Moreover, genes assigned to the biological process “DNA or RNA metabolism” are 4.4-fold overrepresented, and disruption of *MTERF16* (*AT1G78930*) is known to lead to the arrest of embryo development (Babiychuk et al., 2011).

In general, *MTERF* genes in this group respond weakly to physiological perturbations (Figure 3). For example, mRNA expression of *MTERF15* (*AT1G74120*) is naturally induced solely during germination and does not respond to exogenous perturbations. Levels of *MTERF18* (*AT3G60400*) transcripts fall only during germination and upon exposure to heat. It is also noteworthy that 25 of the genes co-expressed with *MTERF* genes in this group code for TPR and PPR proteins. No experimental data on subcellular localization are yet available for 13 of these (AT1G02370, AT1G16480, AT2G16880, AT2G22410 [SLOW GROWTH 1, SLO1], AT2G28050, AT2G33760, AT2G37310, AT2G40720, AT3G13150, AT3G13880, AT3G26540, AT3G49710, and AT4G20090 [EMB1025]). Seven (AT1G09190, AT1G16830, AT2G26790, AT2G37320, AT3G13160, AT4G02750, and AT3G48250) are targeted to mitochondria, and the last mentioned, BUTHIONINE SULFOXIMINE-INSENSITIVE ROOTS 6 (BIR6) has been implicated in splicing of intron 1 of mitochondrial *nad7* transcripts. The PPR proteins AT1G06270, AT1G08070 (ORGANELLE TRANSCRIPT PROCESSING 82, OTP82) and AT1G05750 reside in the chloroplast, and the latter (PIGMENT DEFECTIVE 247, PDE247) is required for editing of *rpoA* and *clpP* chloroplast transcripts (Chateigner-Boutin et al., 2008). The last two, AT1G09220 and AT1G10270, have been localized to the cytosolic ribosome and the nucleus, respectively. The latter (GLUTAMINE RICH PROTEIN 23, GRP23) is essential for early embryogenesis and interacts with RNA polymerase II subunit III (Ding et al., 2006). NUCLEAR ASSEMBLY FACTOR 1 (NAF1, AT1G03530) which binds small nucleolar RNAs, NUCLEAR TATA BOX ASSOCIATED FACTOR II 59 (TAFII59, EMB2781 [AT1G04950])—involved in transcription initiation—and a major component of the nuclear pore complex (NUP155, AT1G14850) also localize to the nucleus. In this context, it is interesting that genes encoding nuclear and mitochondrial DNA repair proteins (AT5G40820, AT1G10520, and AT3G18630) are also co-expressed with *MERF13–19*.

Further mitochondrial representatives are a member of the RCC (regulator of chromosome condensation) family (RCC1/UVR8/GEF-LIKE 3 [RUG3] AT5G60870), which is required for splicing of *nad2* and biogenesis of complex I (Kuhn et al., 2011) and a member of the peptidyl-tRNA hydrolase family (AT5G19830). In addition to the three PPR proteins mentioned above (AT1G06270, AT1G05750, and AT1G08070), the chloroplast fraction includes ORGANELLAR SINGLE-STRANDED DNA BINDING PROTEIN 4 (OSB4, AT1G31010) and a member of the ribosomal protein L1p/L10e family that functions in RNA processing (AT1G06380). Other co-expressed genes encode three RNA-binding proteins predicted to be localized to mitochondria (AT3G46020), the nucleus (AT4G12640), and the cytosol (AT5G08620), respectively. Thus overall, these data indicate a function for mTERF proteins from this group in DNA and RNA metabolism in all three genome-containing compartments, particularly in mitochondria.

### The mitochondrion-associated cluster: mTERF proteins associated with DNA and RNA metabolism in mitochondria, nucleus and the cytoplasm

The set of genes co-expressed with *MTERF20–22* is enriched 4.3-fold for sequences coding for mitochondrial proteins. Moreover, genes for cytosolic and nuclear proteins are also enriched (3.3- and 1.9-fold, respectively) in this set (in the groups so far discussed they are mostly underrepresented), while genes for chloroplast proteins are underrepresented. However, as in group (iii), the biological process “DNA or RNA metabolism” is enriched 5.2-fold. PPR336 (AT1G61870) is known to be associated with mitochondrial polysomes (Uyttewaal et al., 2008) and NUCLEAR FUSION DEFECTIVE 5 (NFD5, AT1G19520) with the nucleus. All other co-expressed TPR and PPR proteins (AT1G15480, AT3G02490, and AT4G02820) are predicted by TargetP (http://www.cbs.dtu.dk/services/TargetP/) and WolF PSORT (http://wolfpsort.org/) to be targeted to both chloroplasts, and mitochondria. Other co-expressed genes coding for organellar proteins in this group that are involved in DNA and RNA metabolism are encoding the mitochondrial RRP4 (RIBOSOMAL RNA PROCESSING 4, AT1G03360), the RNA-binding proteins AT1G06560 (predicted to be localized to mitochondria and chloroplasts) and AT4G40000 (predicted to be mitochondrial) belonging to the NOL1/NOP2/sun family, AT4G13070 (related to the group II intron splicing factor CRS1, and predicted to be localized to mitochondria and chloroplasts) and AT3G06790 (mitochondrial MULTIPLE ORGANELLAR RNA EDITING FACTOR 3; MORF3). Other co-expressed genes encode organellar proteins involved in protein folding, such as AT3G09700 (a chaperone of the DnaJ-domain superfamily), which is predicted to reside in the chloroplast, and AT3G07770 (mitochondrial HEAT SHOCK PROTEIN 89.1). However, a comparable number of co-expressed genes code for cytosolic and especially nuclear proteins involved in (post)-transcriptional and -translational processes. This group includes RRP41 (AT3G61620), a cytosolic 3–5′-exoribonuclease involved in RNA processing, an alba DNA/RNA-binding protein (AT1G20220), a polynucleotidyl transferase involved in DNA repair (AT3G52905) and ABI3-INTERACTING PROTEIN 3 (AIP3, AT1G08780) which is implicated in protein folding and is part of the prefoldin complex. The nuclear proteins represented are AT5G54910 (a DEA(D/H)-box RNA helicase located in the nucleolus), AT1G20580 (a member of a small nuclear ribonucleoprotein family), AT3G24495 (MSH6, a homolog of human MutS involved in DNA mismatch repair) and AT5G24330 (a H3K27 monomethyltransferase required for chromatin structure and gene silencing). Other proteins with a predicted nuclear localization are AT2G02520 (a member of a reverse transcriptase-related family), AT1G20370 (belonging to a family of pseudouridine synthases involved in RNA modification) and the RRM-containing protein AT2G22100.

Thus, the mTERF proteins in this group are also associated with DNA and RNA metabolism in the mitochondria, the nucleus and the cytoplasm. There is as yet no evidence that the nuclear and cytoplasmic components listed here also act in the organelles, e.g., to compensate for organellar defects. Therefore, their associations strongly suggest that mTERF proteins of this group may function directly in the nucleus and/or the cytosol.

### The “others”: expressed at low levels, but may act in the nucleus

All members of this group are represented by very small numbers of ESTs, if any (Figure 2). *MTERF21* (*AT1G56380*) and *MTERF23* (*AT1G62490*) are expressed together, and are co-expressed with six transposable element genes. These observations argue that they may be pseudogenes. However, they are also co-expressed with *AT5G46310* (which codes for a nuclear transcription factor of the WRKY family) and with other genes that code for products predicted to reside in the nucleus. The latter include AT5G18880 (a reverse transcriptase-related protein), AT3G14030 and AT5G62830 (both of which belong to the family of F-box associated ubiquitination effectors) and AT3G49150 (an F-box/RNI-like protein). Strikingly, the set of co-expressed polypeptides includes two other F-box-containing proteins—AT3G52510 (an F-box associated ubiquitination effector protein with a predicted mitochondrial localization) and AT4G26340 (a member of the F-box/RNI-like superfamily with a predicted cytosolic localization). Moreover, just as *MTERF21* and *MTERF23* themselves, all co-expressed genes are by trend low expressed, but have their peak of mRNA expression in pollen.

The product of *MTERF22* (*AT1G62085*; a tandem duplicate) is predicted to be located in mitochondria or chloroplasts. However, attempts to test this using a GFP fusion protein were unsuccessful (Babiychuk et al., 2011). According to CORNET, *MTERF22* is co-expressed (i.e., Pearson correlation coefficient ≥ 0.7) with only two other genes. One of these codes for a protein located in plasmodesmata, and the product of the other is located elsewhere. *MTERF24* (*AT4G19650*; a block duplicate) has a predicted cytosolic or other location but could not be detected as a GFP fusion (Babiychuk et al., 2011). Furthermore, its co-expressed genes have no specified location (Figure 7B, Tables 1 and 2). Additional databases were therefore, mined for co-expression data. The *A. thaliana* Co-Response Database on the CSB.DB web server (http://csbdb.mpimp-golm.mpg.de/csbdb/dbcor/ath.html; Steinhauser et al., 2004) returned no results for either *MTERF22* or *MTERF24*, perhaps because transcripts of the selected genes were below the detection threshold in most of the underlying experiments. This inference is supported by the low EST support of 2 for *MTERF22* (although the transcript was detected in 44.6% of all microarray experiments in Genevestigator) and 8 for *MTERF24* (in Genevestigator the transcript is recorded in only 3% of all microarray datasets). ATTED-II (http://atted.jp/; Obayashi et al., 2009), in which even more microarrays are deposited than in CSB.DB (Usadel et al., 2009) was also queried. In this case, the products of co-expressed genes were predominantly chloroplast and mitochondrial PPR proteins. However, all display only weak co-expression, with correlation coefficients of ≤ 0.5 (in the case of *MTERF22*) and ≤ 0.4 (in the case of *MTERF24*). Altogether, mTERF proteins of this group are weakly expressed. In light of the co-expression profile of *MTERF21* and *MTERF23*, the predicted nuclear localization of mTERF21 (Table 1) and their co-expression with putatively nuclear proteins, mTERF21 and mTERF23 are the most promising candidates for an exclusively nuclear role.

## Concluding remarks

Plant mTERF proteins play an important role in regulating OGE (Meskauskiene et al., 2009; Babiychuk et al., 2011; Quesada et al., 2011) and plant developmental processes, such as gametogenesis (Babiychuk et al., 2011) and embryogenesis (Tzafrir et al., 2004; Meskauskiene et al., 2009; Babiychuk et al., 2011). However, the mechanistic details of their specific functions in these processes have yet to be revealed in plants.

EST and microarray data suggest that the majority of *MTERF* genes in *A. thaliana* are expressed. But, as in the case of the proliferation of PPR proteins in plants (Lurin et al., 2004), it is evident that tens of mTERF proteins are not required for OGE outside the plant kingdom. By analogy with PPR proteins, one may postulate that the expansion of the mTERF family in higher plants allows its members to assume roles that are performed by other proteins in non-plant organisms or to serve functions that are specific to land plants. I favor the latter hypothesis. As sessile organisms, plants can escape neither from biological threats (such as herbivores), nor from unfavorable environmental conditions. The genome plasticity gained through several whole-genome duplications (Sterck et al., 2007), as well as local tandem duplications, or transpositions (Freeling, 2009) has probably played a major part in enabling plants to adapt to environmental changes. Furthermore, the dynamics of genome evolution in plants have led to the expansion of some gene families, including many involved in transcriptional regulation and development (Sterck et al., 2007). Indeed, *A. thaliana MTERF* genes—the duplicated genes in particular—show diverse patterns of mRNA accumulation (see Figures 2 and 3), which supports the idea of the functional diversification of their products. In addition, and in contrast to mTERF proteins in mammals, plant mTERF proteins could have evolved to execute highly specific tasks. Moreover, it can be safely assumed that organelle genomes are gradually accumulating mildly deleterious mutations as a consequence of repressed recombination (Lynch and Blanchard, 1998). The “organelle debugging hypothesis” states that nuclear factors evolved to counteract chloroplast mutations that occurred after the water-to-land transition (Maier et al., 2008). Thus, this hypothesis explains not only the rise of PPR proteins in land plants and accounts for the complexity of chloroplast gene expression (Maier et al., 2008), but perfectly fits the parallel expansion of mTERF proteins.

Whether some of the plant mTERF proteins indeed act as *bona fide* transcription termination factors is not yet clear. It remains to be shown that any of them binds DNA at a specific site like human mTERFs (Jimenez-Menendez et al., 2010; Yakubovskaya et al., 2010). That BSM binds DNA has been demonstrated, but no preferential retention of chloroplast DNA fragments was observed (Babiychuk et al., 2011). Co-expression analysis suggests the involvement of mTERF proteins in almost all stages of OGE. Roles in RNA processing therefore, cannot be ruled out. However, here again, experimental verification of such functions is still lacking.

The data currently to hand suggest that mTERF functions are limited to organelles (Meskauskiene et al., 2009; Babiychuk et al., 2011; Quesada et al., 2011). However, sub-classification of mTERF proteins implies that several plant mTERF proteins might operate in the nucleus and/or the cytosol, or might be localized both to mitochondria and the nucleus. In this respect it is interesting to note that two PPR proteins have been found in the nucleus (Ding et al., 2006; Hammani et al., 2011). GLUTAMINE-RICH PROTEIN 23 (GRP23) interacts with RNA polymerase II and probably functions as a transcriptional regulator essential for early embryogenesis (Ding et al., 2006). In the nucleus PNM1 (for PPR protein localized to the Nucleus and Mitochondria) binds to proteins involved in regulating gene expression, but is also associated with mitochondrial polysomes and may play a role in translation in that organelle (Hammani et al., 2011).

Although bioinformatic analysis of *MTERF* genes and proteins can only suggest tentative functions, it can supply a basis for future genetic and functional studies. Only such experimental approaches can definitively delineate the functions of mTERF proteins, reveal their role in the coordination of the expression of organellar and nuclear genomes, and explain the selective pressures that led to the expansion of *MTERF* genes in higher plant genomes.

### Conflict of interest statement

The author declares that the research was conducted in the absence of any commercial or financial relationships that could be construed as a potential conflict of interest.
